# Changing sex for selfish gain: B chromosomes of Lake Malawi cichlid fish

**DOI:** 10.1038/s41598-019-55774-8

**Published:** 2019-12-27

**Authors:** Frances E. Clark, Thomas D. Kocher

**Affiliations:** Department of Biology, University of Maryland College Park, College Park, MD 20742 USA

**Keywords:** Evolutionary genetics, Evolutionary biology, Genetic linkage study, Evolution, Genetics

## Abstract

B chromosomes are extra, non-essential chromosomes present in addition to the normal complement of A chromosomes. Many species of cichlid fish in Lake Malawi carry a haploid, female-restricted B chromosome. Here we show that this B chromosome exhibits drive, with an average transmission rate of 70%. The offspring of B-transmitting females exhibit a strongly female-biased sex ratio. Genotyping of these offspring reveals the B chromosome carries a female sex determiner that is epistatically dominant to an XY system on linkage group 7. We suggest that this sex determiner evolved to enhance the meiotic drive of the B chromosome. This is some of the first evidence that female meiotic drive can lead to the invasion of new sex chromosomes solely to benefit the driver, and not to compensate for skewed sex ratios.

## Introduction

Every species has its own typical set of chromosomes known as the A chromosomes (As). Many species, across a wide taxonomic range including plants, animals and fungi, also possess one or more additional chromosomes called B chromosomes^1–3^. B chromosomes (Bs) are defined by their presence in some but not all members of a population, and by their non-Mendelian patterns of inheritance^4–6^. The number of Bs can vary among individuals as well as among cells within an individual^1,4^. Their presence and number differs between the gonadal and somatic tissues in several species^1,7^.

B chromosomes are generally considered selfish genetic elements that are parasites of the A genome^4,8^. The two most common phenotypic effects of B chromosomes are on fertility and overall fitness. Fertility typically decreases with the presence of B chromosomes, but this and other effects on fitness are not usually significant unless an individual has many B chromosomes^6,9–11^. Other phenotypic effects of B chromosomes are difficult to perceive (i.e. increased recombination rates, reduced chiasma frequency in males, and increased nondisjunction of A chromosomes) but they are usually detrimental^1,6,9,12,13^. While most Bs probably do have negative effects on their hosts, cases of neutral or even beneficial B chromosomes are known^1,14^. The frequency of B-carriers in a population, their distribution among males and females, and the number of B chromosomes carried by each individual are thought to be the sum of their unusual behavior during mitosis or meiosis, the fitness costs imposed by the B chromosome, and the suppression of B chromosomes by the A genome^2,4,15^.

B chromosomes are frequently univalent, which creates obstacles for proper chromosome segregation in meiosis. This, combined with their non-essential nature, suggests that Bs might have a tendency to be lost quickly. Therefore, special mechanisms are necessary to maintain B chromosomes in populations over evolutionary time. Bs can take advantage of pre-existing meiotic and mitotic machinery to manipulate cell division in their favor and increase their own transmission. Any mechanism that increases transmission above Mendelian expectations is known as drive. Several types of B drive have been discovered in plants, animals and fungi during the last century^1,4,5,16^. However, it has been difficult to elucidate the molecular basis of these drive mechanisms. To date, the most thoroughly described mechanism for B chromosome drive is found in rye^17^.

Due to inherent sex-specific differences in cell division and fertilization, many types of B chromosome drive act only in one sex^1,18^. Furthermore, while a B may drive in one sex, it can also exhibit reduced transmission, or drag, in the other sex^18^. In mealybugs, *Pseudococcus affinis*, the paternally inherited chromosome set is normally eliminated during meiosis leaving the maternally inherited chromosomes to be transmitted. B chromosomes are paternally inherited, but avoid this elimination and are therefore transmitted to >50% of male offspring^4^. The opposite has been found in the zebra finch, *Taeniopygia guttata*, in which drive occurs in females, and males eliminate a haploid B chromosome during meiosis^19,20^.

Most species with B chromosomes show no difference in B-carrier frequency among males and females, but some exceptions are known^1,6^. In the fairy shrimp (*Branchipus schaefferi)*, and one species of characid fish (*Moenkhausia sanctaefilomenae)*, B chromosomes are found exclusively in males^6,21^. In another species of characid (*Astyanax scabripinnis)* they are found more frequently in females^22,23^. In addition, Bs have been shown to influence population sex ratio^6,9,10,24^. It has been suggested that this effect may be the result of B chromosomes interfering with the normal mechanism of sex determination^21,23,25,26^.

B chromosomes can associate with sex chromosomes during meiosis, without altering sex determination, resulting in more frequent transmission to a particular sex. In the grasshopper *Tettigidea lateralis* the B chromosome drives in females, but associates and segregates with the X chromosome during meiosis in males, causing it to be transmitted more frequently to female progeny^1,27^. The opposite has been observed in two other grasshopper species and two species of Hemiptera, where the B chromosome segregates away from the X and is therefore transmitted more frequently to males^28–30^.

Several studies have suggested that B chromosomes have arisen from sex chromosomes, based on the identification of sequences shared between B chromosomes and the sex chromosomes^10,31–34^. However, these sequence similarities may simply reflect the accumulation of similar repetitive elements in the non-recombining portions of both chromosomes. In some of these species, the presence of B chromosomes also coincides with a major karyotypic change involving sex chromosomes, lending further support to the theory that B chromosomes originated from sex chromosomes in these taxa^10,33^. Other studies have proposed that sex chromosomes have arisen from B chromosomes. The Y chromosome of Drosophila shares characteristics commonly associated with B chromosomes. The few genes on the Y, mostly male fertility genes that differ in number and location between the Y’s of different Drosophila species, do not have homologs on the X chromosome. It has been proposed that the Drosophila Y chromosome arose from a B chromosome and is therefore not a degenerated homolog of the X^35^. A study examining the evolutionary origin of the lepidopteran W chromosome concluded that it likely evolved from a B chromosome^36^. The medfly, *Ceratitis capitata*, has an X chromosome polymorphism resulting from the fusion of a B chromosome to the small X chromosome. Males in this species transmit both unattached B chromosomes as well as B-X fusions, while females transmit only the B-X fusions^37^ suggesting this fusion is advantageous for the B chromosome. In each of these examples (fruit flies, butterflies and medflies), the B chromosome-derived portion of the neo-sex chromosome does not carry the sex-determining gene or alter sex determination.

There are only two examples of B chromosomes that have a direct effect on sex determination. In the Lake Victoria cichlid *Lithochromis rubripinnis*, B chromosomes are found only in females. Offspring of a 1B female resulted in 74%, 79% and 91% female progeny, while the offspring of a 2B female were 100% female. The correlation between B chromosome number and sex ratio led to the conclusion that this B is involved in sex determination^9^. In the jewel wasp, *Nasonia vitripennis*, and its close relative, *Trichogramma kaykai*, sex is determined by ploidy. Unfertilized eggs produce haploid males and fertilized eggs produce diploid females. Males, which do not experience meiosis but transmit their entire haploid genome to every sperm cell, typically fertilize more than 50% of available eggs. This results in a female-biased sex ratio and means that males transmit their genetic elements to more than 50% of the next generation. An interesting method of transmission has evolved that ensures transmission of the B solely to males. When a sperm carrying a B chromosome fertilizes an egg, the B chromosome causes chromatin remodeling of the paternal set of A chromosomes resulting in their loss in early mitotic divisions. The B chromosome is the sole remaining paternal chromosome and is incorporated into the maternal haploid set, resulting in a haploid male individual^38,39^.

B chromosomes have been identified in numerous species of cichlid fish in East Africa^9,40–42^. In most species from Lake Victoria, B chromosomes were found in both males and females, and the number of B chromosomes carried ranged from 1–3 among individuals^9,40,41^. So far, *Lithochromis rubripinnis* is the only Lake Victoria species in which B chromosomes have been reported to be limited to females^9^.

In every species of Lake Malawi cichlid with B chromosomes, all B-carriers are female and have a single B chromosome. This distribution of B chromosomes allowed us to rule out several potential drive mechanisms and suggests that this B chromosome drives by preferential segregation in female meiosis^42^. Since the cichlid lineages in lakes Victoria and Malawi diverged several million years ago^43,44^, it is likely that the female-restricted nature of the B chromosomes in the two lakes evolved independently.

In this study, we sought to understand why the Lake Malawi cichlid B chromosome is found only in females. We considered four hypotheses:

Model 1 - The B chromosome pairs and segregates with sex chromosomes in the heterogametic sex. Because Bs are restricted to females, this would require a ZW sex determination system. If the B segregates with the W chromosome, it will be transmitted exclusively to females.

Model 2 - The B chromosome has acquired a feminizing sequence and is itself acting as a sex determiner or W chromosome.

Model 3 - The B chromosome is eliminated early in the development of males.

Model 4 - The B chromosome is lethal in males. Males can inherit the B, but die early in development or before sexual maturity.

Here, we evaluate these hypotheses by quantifying the transmission of B chromosomes in a series of genetic crosses in the Lake Malawi cichlid, *Metriaclima lombardoi*.

## Materials and Methods

### Animals

All procedures involving live fish were approved by and conducted in accordance with the University of Maryland IACUC under Protocol #R-10-73. At least seven species of Lake Malawi cichlids carry B chromosomes^42^. *Metriaclima lombardoi*, a species in which approximately 11% of females carry a B chromosome, was used for this study. Live, wild-caught individuals were imported from Lake Malawi, Africa in 2014, 2015 and 2016 to establish a colony in the Tropical Aquaculture Facility at the University of Maryland.

### Crosses

Following our previous methods^42^, we used PCR to determine whether individual fish carried a B chromosome. Females with a B chromosome were crossed to males lacking a B. The parents of each cross differ, however the B females of this laboratory line are all descendants of just 3 B female founders. As a result, the B females in several of these crosses are related to one another (sisters, aunts/nieces, cousins). For comparison to the B chromosome cross, females without a B (NoB females) were crossed to males lacking a B. Lake Malawi cichlids are maternal mouth brooders, holding the offspring in their mouth for 2-3 weeks. Offspring were collected from the female at 1 week and raised together in a tank. A fin clip was taken from the brooding female at the time the offspring were collected.

Each family was sampled after reaching sexual maturity (approximately 9 months). While some amount of mortality in those 9 months is typical for cichlids, difficulties establishing the laboratory line (mainly bacterial infections) led to high and variable mortality rates and we strongly encourage caution when interpreting the mortality rates in our data (Supplementary Table S1). Individuals were euthanized with tricaine methanesulfonate (MS-222) and inspected for testes or ovaries to confirm sex. *M. lombardoi* is thought to have bright yellow males and bright blue females, but we found several yellow females and blue males as well as many individuals equally blue and yellow. This was true for both B and NoB individuals and there is no evidence B chromosome presence is responsible for differences in color. For this reason, we did not use color as an indicator of sex and strongly discourage this method for sexing *M. lombardoi*. Fin clips were collected from each individual for subsequent genotyping of sex-linked markers.

### Genotyping

DNA was extracted from fin tissue using standard phenol chloroform methods and phase-lock gel tubes (5prime, Gaithersburg, MD, USA). The B-specific primers described in^42^ were used to genotype for B presence/absence. All individuals were amplified with the B-specific primer set for Seq. 32 as well as the SWS1 (UV) opsin primer set to confirm that the quality of the DNA was sufficient for amplification. If a sample showed poor amplification of the opsin primer set we did not attempt to score presence/absence of the B chromosome. Samples with clear amplification of the opsin primer set but ambiguous amplification of the Seq. 32 primer set were then amplified with the remaining four B-specific primer sets to score presence/absence of the B chromosome.

Several sex-determining systems have been found among cichlids^45^. An XY system on linkage group 7 (LG7) and a WZ system on linkage group 5 (LG5) have been previously documented in several species of *Metriclima* from Lake Malawi^46^. To confirm which sex determination system(s) is acting in *M. lombardoi*, families were examined for linkage between phenotypic sex and microsatellite markers for these two known sex-determination systems. We used markers on LG5 (UNH2139 and c-Ski) and LG7 (UNH2086 and UNH2031)^46^. After PCR amplification of these microsatellite markers with fluorescently labeled primers, we separated the fragments on an Applied Biosystems 377 DNA sequencer and analyzed allele sizes with GeneScan v3.1.2.

Sex ratios, reported as male frequency, were analyzed for statistical significance with a binomial test. B transmission, reported as B-carrier frequency, was also analyzed with a binomial test. A Fisher’s exact test was performed on each family to assess the relationship between B chromosome presence and sex. Finally, to assess the relationship between B chromosome presence and sex while accounting for family identity, we constructed generalized linear mixed-effect models. Models were constructed with and without B chromosome presence as a variable and analyzed with the glmer function in the lme4 package for R. Because the response variable, sex, is binary, a binomial distribution was used with a logit link function. To evaluate the significance of the B chromosome variable, these models were compared with a likelihood ratio test using the anova function in R. The generalized linear mixed-effect models were as follows:

$$Effect\_on\_sex.null=sex \sim haplotyp{e}_{paternal}+(1|family)$$

$$Effect\_on\_sex.model=sex \sim haplotyp{e}_{paternal}+{B}_{presence/absence}+(1|family)$$

## Results

### Transmission of the B chromosome

The six families resulting from a “B cross” (a B female crossed with a NoB male) showed B transmission ranging from 50% to 95% (Table 1). Although five of the six families had B transmission rates greater than 50%, only two of these families (A008 and A040) had a statistically significant (p-value ≤ 0.05) deviation from Mendelian expectations. The average transmission rate among these six B families is 67%. Another binomial test, assuming this averaged transmission rate, was performed on each family and five families were statistically consistent with a transmission rate of 67%. The remaining family, A008 represented a statistically significant deviation from even a 67% transmission rate (p-value = 0.00698) and was only statistically consistent with transmission rates greater than 75%. While a mechanism of drive has already been proposed for the B chromosome of this species, this is the first evidence that drive does indeed occur.

**Table 1:** Transmission of B chromosomes in six families of *Metriaclima lombardoi*.

B Family	# of B individuals	# of NoB individuals	B transmission rate	Binomial p-value
A008	19	1	0.95	0.00004**
A018	16	10	0.62	0.32690
A035	8	8	0.50	1.00000
A036	18	11	0.62	0.26490
A038	14	10	0.58	0.54130
A040	29	9	0.76	0.00166**

PCR was used to determine whether the individuals in each family carried a B. The B transmission rate in each family is reported as the proportion of B individuals. A binomial test was performed, and families A008 and A040 showed significant (p-value ≤ 0.05) deviations from the Mendelian expectation of 50% transmission.

### Sex ratios of families

**Figure 1:**
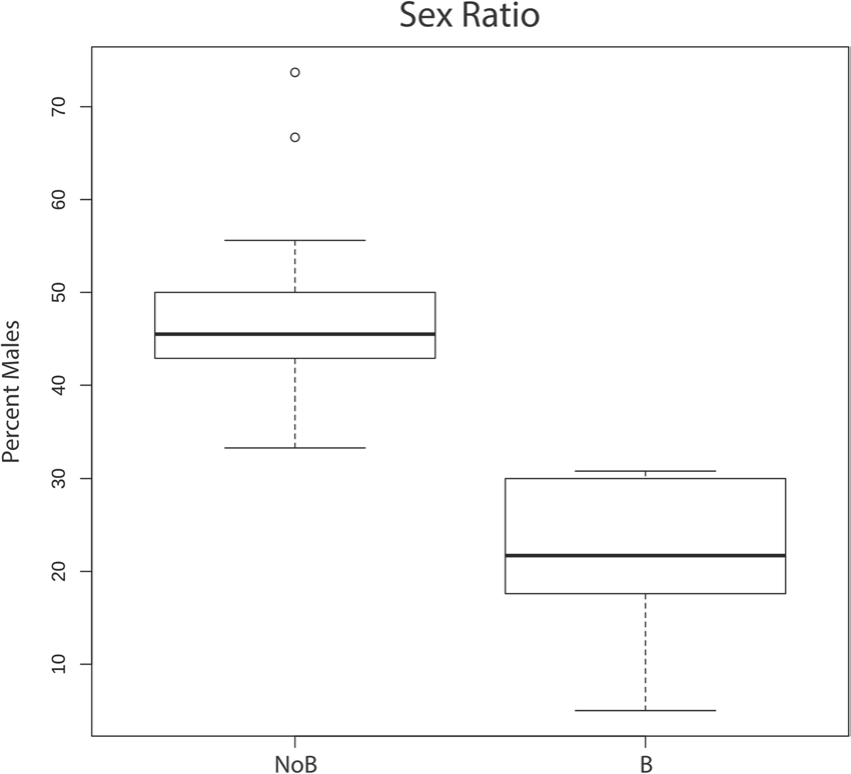
Sex ratio in NoB and B families. Box plots are shown for the sex ratio, reported as percent of males, for 15 NoB families (N = 184) and 5 B families (N = 93).

The sex ratio among 15 families resulting from NoB crosses averaged 0.48 (or 48% males) with a median of 0.45 (or 45% males) as shown in Table 2 and Fig. 1. Only 5 of the 6 families resulting from a B cross had sufficient data on sex to analyze. The sex ratio for these families ranged from 0.05 to 0.31 (Table 3). Three of these families (A008, A018, A038) were statistically different from an even sex ratio according to a binomial test. A Fisher’s exact test was performed on each family to further confirm the association between B chromosome presence and sex (Table 4) and families A008, A018, A036 and A038 had a significant p-value (p-value ≤ 0.05). The transmission of this B chromosome is skewing the sex ratio towards females. The average sex ratio among the B families was approximately 20% males. Another binomial test performed assuming a sex ratio of 0.2 showed that each B family was statistically consistent with a sex ratio of 0.2. These results allow us to reject two hypotheses, segregation with a W chromosome and B chromosome elimination in males, as these are not expected to alter sex ratio. Additional analyses were conducted to further distinguish the two remaining hypotheses: B sex determination and male lethality.

**Table 2:** NoB family sex ratio in fifteen families of *Metriaclima lombardoi*.

NoB Family	Initial # of progeny	# of confirmed males	# of confirmed females	Sex Ratio
A003	35	3	4	42.9
A006	39	5	8	38.5
A009	36	5	6	45.5
A011	35	7	9	43.8
A013	48	4	2	66.7
A017	45	2	4	33.3
A022	64	5	5	50.0
A024	39	3	3	50.0
A026	31	5	4	55.6
A027	43	9	12	42.9
A028	40	10	11	47.6
A029	15	4	6	40.0
A032	42	5	6	45.5
A033	29	14	5	73.7
A034	53	9	9	50.0
		Total: 90	Total: 94	Mean: 48.4

Sex ratio is reported as the percentage of male progeny. Sex was determined by dissection and inspection of the gonads. Only individuals surviving to sexual maturity could be confirmed as male or female.

**Table 3:** B family sex ratio in five families of *Metriaclima lombardoi* segregating B chromosomes.

B Family	# of Males	# of Females	Sex Ratio	Binomial p-value
A008	1	19	0.05	0.00004**
A018	3	14	0.18	0.01273*
A035	4	9	0.31	0.26680
A036	6	14	0.30	0.11530
A038	5	18	0.22	0.01062*

Sex ratio is reported as the percentage of male progeny. A binomial test, assuming a sex ratio of 0.5, was performed on each family. Families A008, A018 and A038 significantly (p-value ≤ 0.05) deviated from the expected 0.5 sex ratio with a bias towards females. Sex was determined by dissection and inspection of the gonads. Only individuals surviving to sexual maturity could be confirmed as male or female.

**Table 4:** Association between B chromosome and sex.

B Family	# of NoB Males	# of NoB Females	# of B Males	#of B Females	Fishers exact p-value
A008	1	0	0	19	0.05000*
A018	3	1	0	13	0.00588**
A035	4	4	0	5	0.10490
A036	6	3	0	11	0.00217**
A038	5	5	0	13	0.00749**

The association between B chromosome presence and sex ratio is evaluated with a Fisher’s exact test for each family. Families A008, A018, A036 and A038 had a significant p-value (p-value ≤ 0.05), supporting an association between B chromosomes and the female sex.

### Sex linkage

**Figure 2:**
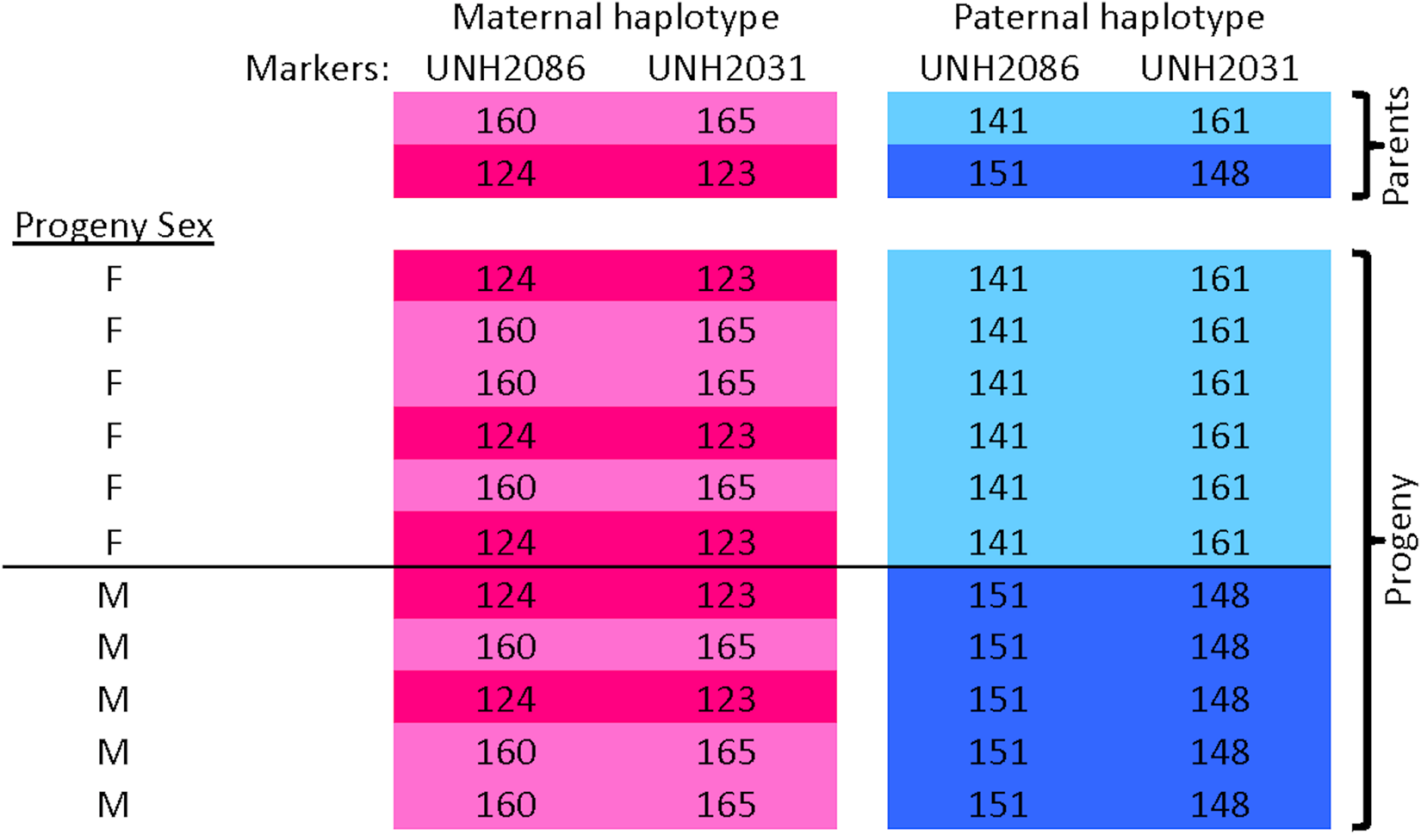
Sex-linkage of a *Metriaclima lombardoi* family (A009) lacking B chromosomes. The allele sizes of two microsatellite markers (UNH2086 and UNH2031) known to be tightly linked with the LG7 XY sex determination system^46^ are shown. The parental haplotypes are shown above those of the offspring. The haplotypes inherited from the mother (dam) are shown in light and dark pink while the haplotypes inherited from the father (sire) are shown in light and dark blue. All female offspring inherited one haplotype (light blue) from their father, while all male offspring inherited the other haplotype (dark blue) from their father. This is consistent with linkage to an XY sex determination system where the light blue haplotype is tightly linked with the X allele and the dark blue haplotype is tightly linked with the Y allele. Please note, only individuals of the family for which both sex and genotype data was obtained are included in this figure. Individuals missing one or both of these data types were excluded for simplicity.

Families resulting from a NoB cross revealed tight linkage with the LG7 XY system (Fig. 2, Supplementary Figure S1), consistent with earlier findings^46^. All females inherited the same haplotype (depicted as light blue in Fig. 2) from the father while all males inherited the other haplotype (dark blue). This is consistent with an XY sex determination system on LG7.

**Figure 3:**
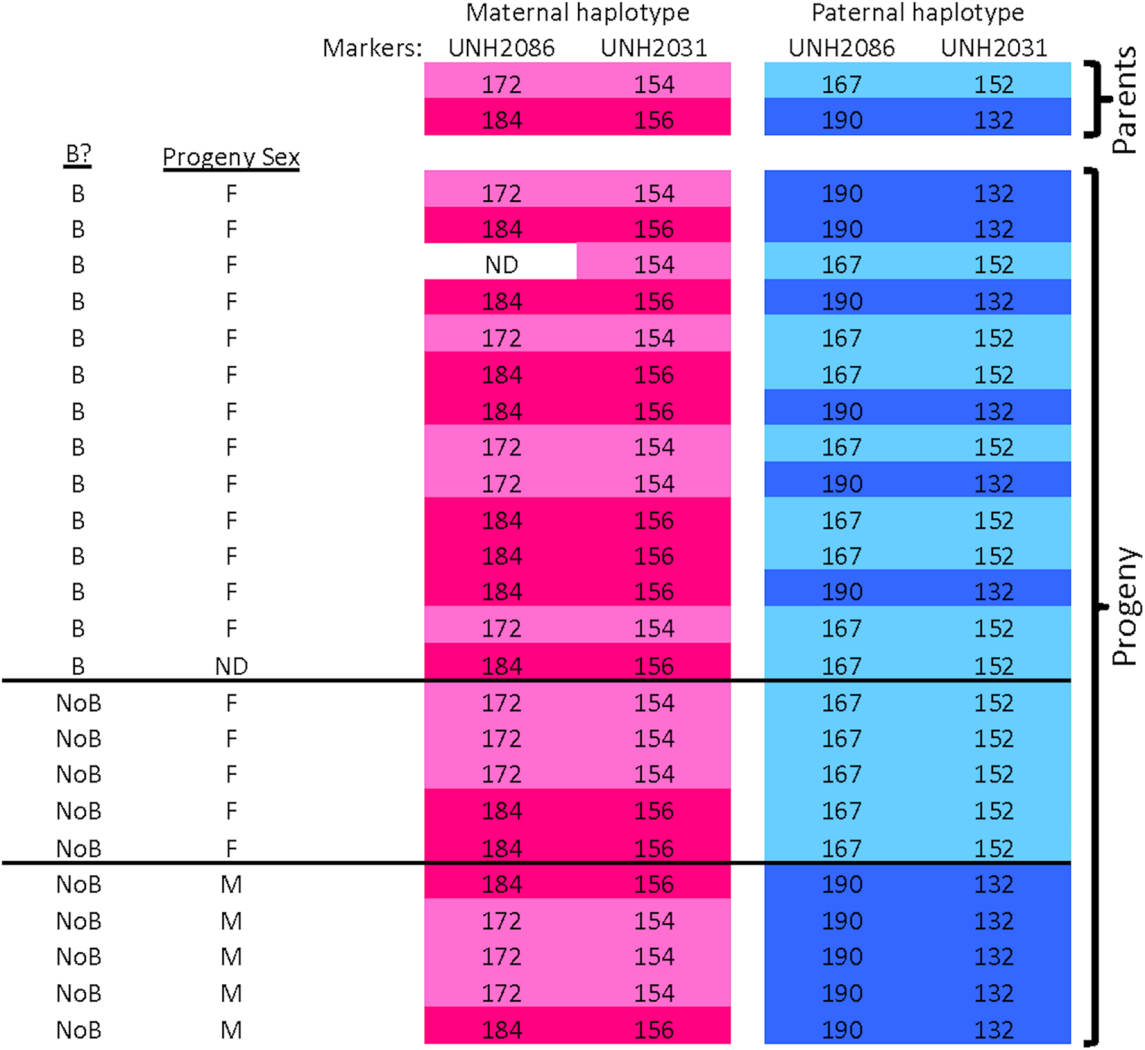
Sex-linkage in a family (A038) with B chromosomes. The allele sizes of two microsatellite markers (UNH2086 and UNH2031) known to be tightly linked with the LG7 XY sex determination system^46^ are shown. The parental haplotypes are shown above those of the offspring. The haplotypes inherited from the mother (dam) are shown in light and dark pink while the haplotypes inherited from the father (sire) are shown in light and dark blue. Among NoB progeny, sex is linked to this region, as evidenced by all NoB female progeny having inherited one paternal haplotype (light blue) and all NoB male progeny having inherited the other haplotype (dark blue). However, this linkage does not exist in the progeny with a B chromosome. Please note, only NoB individuals of the family for which sex and genotype data was obtained or B individuals for which genotype data was obtained are included in this figure. NoB individuals missing one or both of these data types, and B individuals missing genotype data were excluded.

Families resulting from a B cross revealed linkage with the LG7 XY only among NoB individuals. The B individuals could possess either the X or Y paternal haplotype in this region (Fig. 3, Supplementary Figure S1). These linkage data demonstrate that most families of *M. lombardoi* segregate an XY sex determination system on LG7. However, the sex of the B individuals is not determined by the LG7 locus alone. Generalized linear mixed-effects models were created, with and without B presence as a variable, and compared with a likelihood ratio test to evaluate the importance of the B chromosome for determining sex. Individuals of 3 NoB families and 5 B families for which genotype and sex were known (N = 123) were used to investigate these models. B chromosome presence affected the ability to predict sex in a statistically significant manner (X^2^ (1) = 71.163, p < 2.2*10^−16^). The B chromosome is epistatically dominant to the Y haplotype, causing a female phenotype. In conclusion, the female-limited distribution of the Lake Malawi cichlid B is the result of an acquired feminizing sex determiner on the B chromosome.

## Discussion

This study provides evidence that the B chromosome of Lake Malawi cichlids experiences drive in females. While only two of the six B chromosome families provided statistically significant evidence for drive, the remaining four families were statistically consistent with the averaged transmission rate of 67%. There appears to be variation in the strength of this drive among individuals, similar to reports in characiform fish^47^, grasshoppers^48–51^, daisies^52^, rye^53–55^, and the mealybug^56–58^. As found in the mealybug, this variation may be the result of polymorphic loci in the A genome that suppress drive.

There are numerous mechanisms by which B chromosome drive might be accomplished^1,4,18^. We previously argued that there are only two forms of drive involving nondisjunction and/or preferential segregation that would produce a population where B-carriers consistently had a single B^42^. The first, nondisjunction and preferential segregation during a pre-meiotic mitosis, would require an unlikely asymmetric mitotic division prior to formation of the germline. The second, preferential segregation during meiosis I, is more likely because it has fewer requirements and has already been documented in several other taxa^4,18,48,52,56,59–67^. Drive via preferential segregation in meiosis I is only effective in individuals which have asymmetric meiotic divisions. As a result, this B would drive in females but not in males.

If this B chromosome acquired a mechanism to ensure it was transmitted solely to females, it would increase the fitness of the B chromosome because it would increase the opportunity for drive from 50% to 100%. In this study, we evaluated 4 hypotheses consistent with a female-limited B presence. Two of these hypotheses, segregation with a W chromosome and a feminizing sex determiner on the B, would result in transmission solely to females, increasing B fitness. The other two hypotheses would involve transmission of the B to males, but either subsequent loss of the B or death of the male, neither of which would increase the fitness of the B chromosome.

Our results demonstrate that the B chromosome in *Metriaclima lombardoi* is acting as a feminizing sex determiner, or univalent W chromosome, that is epistatically dominant to the Y chromosome on linkage group 7. As a result, XY-WO, XX-WO and XX-OO individuals are female and XY-OO individuals are male. Since B chromosomes are generally thought to have little impact on phenotype, this is a noteworthy phenotypic effect of a B chromosome. Furthermore, this WO/OO system is remarkably similar to that of the New Zealand frog, *Leiopelma hochstetteri*^68,69^. Sharbel and his colleagues proposed that the many B chromosomes seen in this frog originated through sex chromosome devolution where the W acquired a loss-of-function mutation^33^. Yoshida and his colleagues counter-proposed that the W chromosome may have evolved from one of the many B chromosomes that acquired a feminizing sex determiner^9^. While these alternative hypotheses each remain viable, we submit the Lake Malawi cichlid B chromosome as a second example of a univalent W chromosome evolving from a B chromosome, lending further credibility to the latter hypothesis.

Female meiotic drive has previously been suggested to be a force contributing to the evolution of sex chromosomes. This might occur through the selective fixation of X-autosome fusions^70^, to re-establish a Fisherian sex ratio skewed by sex chromosome meiotic drive^71,72^, or to resolve genetic conflict produced by the sexually antagonistic alleles selectively acquired by sex-specific drivers^73,74^. The literature on female meiotic drive does not, to our knowledge, discuss the evolution of sex determination to increase driver fitness.

This study is perhaps the first clear example of female meiotic drive leading to the evolution of a dominant sex chromosome strictly by selfish mechanisms. The new sex determiner gives the B chromosome a selective advantage by enhancing the opportunity for drive. B chromosomes that drive only in males might similarly lead to the evolution of new dominant male sex determiners.

Several B chromosomes with known drive mechanisms have been correlated with changes in sex ratio, and B chromosomes with unknown drive mechanisms have been shown to be sex determiners^9^. But in each case, data on both aspects, the mechanism of drive and the effect on sex determination, were not simultaneously available. Indeed, Yoshida and colleagues proposed the Lake Victoria cichlid B chromosome gained its role as a sex determiner as a result of sexually antagonistic loci located on the B chromosome. However, future characterization of the mechanism of drive in cichlid species from Lake Victoria may offer a different perspective.

The B chromosomes of the wasps *Nasonia vitripennis* and *Trichogramma kaykai*, are thought to have gained their role as a sex determiner either at or shortly after the origin of the B chromosome. Since these B chromosomes might experience high levels of transmission drag in females^38,75–77^, this chromosome would have to quickly evolve a mechanism to reduce drag in order to be maintained in the population. The sex determination effect allows the B chromosome to avoid female drag while gaining drive through high male fertility rates. Elimination of the paternal chromosome set to masculinize the embryo is simultaneously the mechanism of sex determination and the mechanism of drive^39^. While this example of drive-associated sex determination did not evolve in response to female meiotic drive, but possibly in order to avoid female meiotic drag, the similarities in the underlying evolutionary forces are striking.

Cichlids have one of the highest rates of sex chromosome turnover known in vertebrates. Already, more than a dozen sex determination systems have been identified among East African cichlids^45^. Theory suggests that sex chromosome turnover can be fueled by sexually antagonistic selection on alleles genetically linked to a sex determiner^78,79^. Another hypothesis is that meiotic drive distorts the segregation of sex chromosomes, altering the sex ratio of the population, which might lead to the invasion of a new sex determiner to restore an even sex ratio^71^.

We suggest that B chromosomes may contribute to the high rate of sex chromosome replacement observed in African cichlids. The B chromosome carries an epistatically dominant feminizing allele that is altering the sex ratio of the population for selfish gain. The altered sex ratio likely increases selection for a masculinizing sex determiner in the A genome. There is therefore the potential for a Red Queen dynamic. B chromosome drive in females leads to the evolution of a dominant female sex determiner that benefits the B chromosome but alters the sex ratio. This in turns selects for new dominant male sex determiners that benefit the A genome by restoring the Fisherian sex ratio and decreasing the fitness of the B chromosome. We imagine these forces might promote a cycle of sex chromosome turnover in these lineages.

## Supplementary information

Supplementary Information

## Data Availability

All data generated or analyzed during this study are included in this manuscript and its Supplementary Information files.
