# Supplementary material for: Changing sex for selfish gain: B chromosomes of Lake Malawi cichlid fish
Source: Sci Rep. 2019 Dec 27;9:20213. doi: 10.1038/s41598-019-55774-8 (PMC6934658; doi:10.1038/s41598-019-55774-8)
Supplement: Supplementary file 1 — Supplementary Information [file 41598_2019_55774_MOESM1_ESM.pdf]

Changing sex for selfish gain: B chromosomes of Lake Malawi cichlid fish
Clark and Kocher

Family	Cross type	Initial # of progeny	# with recorded B/NoB genotype	# with recorded sex
A008	B	32	20	20
A018	B	43	26	17
A035	B	17	16	13
A036	B	41	29	20
A038	B	35	24	23
A040	B	41	38	0
A003	NoB	35	-	7
A006	NoB	39	-	13
A009	NoB	36	-	11
A011	NoB	35	-	16
A013	NoB	48	-	6
A017	NoB	45	-	6
A022	NoB	64	-	10
A024	NoB	39	-	6
A026	NoB	31	-	9
A027	NoB	43	-	21
A028	NoB	40	-	21
A029	NoB	15	-	10
A032	NoB	42	-	11
A033	NoB	29	-	19
A034	NoB	53	-	18

Supplementary Table S1. Sample Information. The 21 *Metriaclima lombardoi* families used in this study are listed with information regarding cross type, family size and the data collected from each family. The number of individuals initially collected from the mother (dam) for each family is reported in the 3rd column. Individuals were euthanized after reaching sexual maturity (approximately 9 months) and inspected for testes or ovaries to confirm sex. Fin tissue was collected either after euthanasia or discovery of early mortality. Not all fin tissue collected after early mortality yielded viable DNA. Mortality prior to sexual maturation resulted in individuals for which sex could not be determined. The 4th and 5th columns show the number of individuals of each family for which genotype (B presence or absence) and sex was determined, respectively. Please note, difficulties establishing this laboratory line (mainly bacterial infections) led to high and variable mortality rates. Interpretation of any correlation between mortality rates and other factors (such as B chromosome presence) is therefore likely confounded and misleading.

Supplementary Figure S1. Sex-linkage for nine families (six families with B chromosomes and 3 families without B chromosomes). The families from Figures 2 and 3 are included in this figure. The allele sizes of two microsatellite markers (UNH2086 and UNH2031) known to be tightly linked with the LG7 XY sex determination system [46] are shown. For each family, the parental haplotypes are shown above those of the offspring. The haplotypes inherited from the mother (dam) are shown in light and dark pink while the haplotypes inherited from the father (sire) are shown in light and dark blue. Gray indicates that the two alleles of the parent were of identical size and could not be differentiated. The letters ND indicate “no data” where quality data could not be obtained. Sex, sample ID and genotype are reported for progeny of NoB families. Presence or absence of a B chromosome, sex, sample ID and genotype are reported for progeny of B families, with the exception of family A040 where sex was not able to be determined due to premature death of all individuals. Please note, only NoB individuals of each family for which sex and genotype data was obtained or B individuals for which genotype data was obtained are included in this figure. NoB individuals missing one or both of these data types, and B individuals missing genotype data were excluded for simplicity.

B Families

Family: A008

				Maternal haplotype		Paternal haplotype	
			Markers:	UNH2086	UNH2031	UNH2086	UNH2031
				151	175	153	147
				184	150	140	160
B?	Sex	Sample ID					
B	F	2015-1166		184	ND	153	ND
B	F	2015-1167		151	175	153	147
B	F	2016-1010		184	150	140	160
B	F	2016-1012		151	175	153	147
B	F	2016-1013		184	150	153	147
B	F	2016-1014		184	150	140	160
B	F	2016-1015		151	175	153	147
B	F	2016-1016		184	150	153	147
B	F	2016-1017		184	150	153	147
B	F	2016-1018		184	150	153	147
B	F	2016-1019		151	175	153	147
B	F	2016-1020		151	175	140	160
B	F	2016-1021		184	150	140	160
B	F	2016-1022		184	150	140	160
B	F	2016-1023		184	150	140	160
B	F	2016-1024		151	ND	140	ND
B	F	2016-1025		184	150	140	160
B	F	2016-1026		184	150	140	160
B	F	2016-1027		151	175	140	160
NoB	M	2016-1011		151	175	140	160

Family: A018

				Maternal haplotype		Paternal haplotype	
			Markers:	UNH2086	UNH2031	UNH2086	UNH2031
				158	158	172	154
				184	156	170	154
B?	Sex	Sample ID					
B	F	2016-1521		184	156	170	154
B	F	2016-1526		158	ND	172	154
B	F	2016-1530		184	156	170	154
B	F	2016-1531		158	158	172	154
B	F	2016-1534		184	156	170	154
B	F	2016-1540		158	158	172	154
B	F	2016-1541		184	156	172	154
B	F	2016-1542		184	156	172	154
B	F	2016-1544		184	156	172	154
B	F	2016-1547		158	158	170	154
B	F	2017-1002		158	ND	172	154
B	F	2016-1524		184	ND	170	154
B	F	2016-1525		158	ND	172	154
B	ND	2016-1527		184	156	170	154
B	ND	2016-1543		184	156	172	154
B	ND	2016-1330		158	158	172	154
NoB	F	2016-1522		184	156	172	154
NoB	M	2016-1528		158	158	170	154
NoB	M	2016-1533		184	156	170	154
NoB	M	2016-1538		184	156	170	154

Family: A035

B?	Sex	Sample ID	Maternal haplotype UNH2086	Maternal haplotype UNH2031	Paternal haplotype UNH2086	Paternal haplotype UNH2031
		Markers:	UNH2086	UNH2031	UNH2086	UNH2031
			158	158	188	162
			172	153	154	149
B	F	2017-1037	172	153	188	162
B	F	2017-1042	158	158	154	149
B	F	2017-1043	172	153	188	162
B	F	2017-1044	172	153	188	162
B	F	2017-1045	158	158	154	149
B	ND	2018-1003	172	153	188	162
B	ND	2018-1004	172	153	154	149
NoB	F	2017-1036	172	153	188	162
NoB	F	2017-1041	172	153	188	162
NoB	F	2017-1040	158	158	188	162
NoB	M	2017-1034	158	153	154	149
NoB	M	2017-1035	158	158	154	149
NoB	M	2017-1038	172	153	154	149
NoB	M	2017-1039	172	153	154	149

Family: A036

B?	Sex	Sample ID	Maternal haplotype UNH2086	Maternal haplotype UNH2031	Paternal haplotype UNH2086	Paternal haplotype UNH2031
		Markers:	UNH2086	UNH2031	UNH2086	UNH2031
			170	156	188	162
			184	156	154	149
B	F	2018-1087	184	156	154	149
B	F	2018-1088	170	156	188	162
B	F	2018-1089	184	156	188	162
B	F	2018-1231	170	156	188	162
B	F	2018-1232	170	156	154	149
B	F	2018-1233	170	156	188	162
B	F	2018-1234	184	156	188	162
B	F	2018-1235	184	156	154	149
B	F	2018-1236	170	156	188	162
B	F	2018-1237	170	156	188	162
B	F	2018-1238	ND	156	ND	162
B	ND	2017-1013	170	156	154	149
B	ND	2018-1037	170	156	188	162
B	ND	2018-1039	184	156	154	149
B	ND	2018-1040	170	156	154	149
NoB	F	2018-1030	184	156	188	162
NoB	F	2018-1071	184	156	188	162
NoB	F	2018-1073	170	156	188	162
NoB	M	2018-1029	170	156	154	149
NoB	M	2018-1082	170	156	154	149
NoB	M	2018-1083	170	156	154	149
NoB	M	2018-1084	184	156	154	149
NoB	M	2018-1085	170	156	154	149
NoB	M	2018-1086	184	156	154	149

Family: A038

				Maternal haplotype		Paternal haplotype	
			Markers:	UNH2086	UNH2031	UNH2086	UNH2031
				172	154	167	152
				184	156	190	132
B?	Sex	Sample ID					
B	F	2018-1211		172	154	190	132
B	F	2018-1212		184	156	190	132
B	F	2018-1215		ND	154	167	152
B	F	2018-1216		184	156	190	132
B	F	2018-1217		172	154	167	152
B	F	2018-1218		184	156	167	152
B	F	2018-1220		184	156	190	132
B	F	2018-1225		172	154	167	152
B	F	2018-1227		172	154	190	132
B	F	2018-1228		184	156	167	152
B	F	2018-1230		184	156	167	152
B	F	2018-1229		184	156	190	132
B	F	2018-1226		172	154	167	152
B	ND	2017-1056		184	156	167	152
NoB	F	2018-1223		172	154	167	152
NoB	F	2018-1224		172	154	167	152
NoB	F	2018-1214		172	154	167	152
NoB	F	2018-1221		184	156	167	152
NoB	F	2018-1222		184	156	167	152
NoB	M	2018-1075		184	156	190	132
NoB	M	2018-1210		172	154	190	132
NoB	M	2018-1213		172	154	190	132
NoB	M	2018-1219		172	154	190	132
NoB	M	2018-1077		184	156	190	132

Family: A040

B?	Sample ID		Maternal haplotype		Paternal haplotype	
		Markers:	UNH2086	UNH2031	UNH2086	UNH2031
			172	156	167	152
			184	156	190	132
B?	Sample ID					
B	2018-1206		184	156	190	132
B	2018-1251		172	156	167	152
B	2018-1252		184	156	167	152
B	2018-1300		172	156	190	132
B	2019-1001		172	156	190	132
B	2018-1041		184	156	167	152
B	2018-1042		184	156	190	132
B	2018-1044		184	156	190	132
B	2018-1046		172	156	167	152
B	2018-1047		184	156	190	132
B	2018-1048		172	156	167	152
B	2018-1049		184	156	190	132
B	2018-1050		172	156	190	132
B	2018-1052		172	156	167	152
B	2018-1053		184	156	167	152
B	2018-1055		172	156	190	132
B	2018-1056		172	156	167	152
B	2018-1057		184	156	190	132
B	2018-1058		172	156	190	132
B	2018-1060		172	156	167	152
B	2018-1061		172	156	167	152
B	2018-1063		184	156	167	152
B	2018-1064		184	156	167	152
B	2018-1065		172	156	190	132
B	2018-1066		172	156	190	132
B	2018-1067		184	156	167	152
B	2018-1068		184	156	167	152
B	2018-1069		184	156	167	152
B	2018-1070		184	156	167	152
NoB	2018-1264		ND	ND	167	ND
NoB	2018-1299		184	156	167	152
NoB	2018-1239		184	156	190	132
NoB	2018-1043		184	156	167	152
NoB	2018-1045		184	156	190	132
NoB	2018-1051		184	156	167	152
NoB	2018-1054		172	156	190	132
NoB	2018-1059		184	156	167	152
NoB	2018-1062		184	156	190	132

NoB Families

Family: A003

Sex	Sample ID	Maternal haplotype UNH2086	Maternal haplotype UNH2031	Paternal haplotype UNH2086	Paternal haplotype UNH2031
	Markers:	UNH2086	UNH2031	UNH2086	UNH2031
		167	190	169	156
		157	175	159	129
F	2015-1169	167	190	169	156
F	2015-1170	167	190	169	156
F	2015-1171	157	175	169	156
F	2015-1172	167	190	169	156
M	2015-1173	157	175	159	129
M	2015-1174	157	175	159	129
M	2015-1175	167	190	159	129

Family: A006

Sex	Sample ID	Maternal haplotype UNH2086	Maternal haplotype UNH2031	Paternal haplotype UNH2086	Paternal haplotype UNH2031
	Markers:	UNH2086	UNH2031	UNH2086	UNH2031
		184	150	142	160
		187	160	153	148
F	2015-1177	187	160	142	160
F	2015-1178	184	150	142	160
F	2016-1001	184	150	142	160
F	2016-1002	184	150	142	160
F	2016-1003	184	150	142	160
F	2016-1004	184	150	142	160
F	2016-1006	184	150	142	160
F	2016-1007	184	150	142	160
M	2015-1176	184	150	153	148
M	2015-1179	184	150	153	148
M	2015-1180	184	150	153	148
M	2016-1005	187	160	153	148
M	2016-1009	184	150	153	148

Family: A009

Sex	Sample ID	Maternal haplotype UNH2086	Maternal haplotype UNH2031	Paternal haplotype UNH2086	Paternal haplotype UNH2031
	Markers:	UNH2086	UNH2031	UNH2086	UNH2031
		160	165	141	161
		124	123	151	148
F	2016-1033	124	123	141	161
F	2016-1034	160	165	141	161
F	2016-1035	160	165	141	161
F	2016-1036	124	123	141	161
F	2016-1037	160	165	141	161
F	2016-1038	124	123	141	161
M	2016-1028	124	123	151	148
M	2016-1029	160	165	151	148
M	2016-1030	124	123	151	148
M	2016-1031	160	165	151	148
M	2016-1032	160	165	151	148
